# Macroid Formation in *Salmacina stellaebayensis* n. sp. From Mauritania's Baie de l'Étoile With New Insights on Mitogenome Evolution in Serpulidae (Annelida)

**DOI:** 10.1002/ece3.73016

**Published:** 2026-01-29

**Authors:** Hannah A. Cremer, Elena Kupriyanova, Alexander H. Knorrn, Sidi M. M. Moctar, Andre Freiwald, Ekin Tilic

**Affiliations:** ^1^ Senckenberg Research Institute and Natural History Museum Frankfurt Senckenberg—Leibniz Institution for Biodiversity and Earth System Research Frankfurt am Main Germany; ^2^ Australian Museum Research Institute Australian Museum Sydney New South Wales Australia; ^3^ Senckenberg—Leibniz Institution for Biodiversity and Earth System Research Wilhelmshaven Germany; ^4^ Institut Mauritanien de Recherche Océanographique et Des Pêches Nouadhibou Mauritania

**Keywords:** Filograna, NE Atlantic, phylogenetics, polychaeta, taxonomy

## Abstract

Knowledge of serpulid diversity along the West African coast remains scarce. Here we describe *Salmacina stellaebayensis* n. sp. from the Baie de l'Étoile, Mauritania, a new macroid‐former generating striking star‐shaped pseudo‐colonies. Combining classical morphology with genomic data, we provide the first complete mitochondrial genome for the genus *Salmacina* and recover additional nuclear markers to clarify its phylogenetic placement within Serpulidae. *Filograna* and *Salmacina* form a taxonomically challenging group with uncertain generic boundaries. While additional data from operculate *Filograna* are needed, this study provides a valuable baseline and much‐needed molecular framework for future revisions. Comparative analyses of 17 serpulid mitogenomes reveal extensive variation in gene order, supporting the view that mitogenome plasticity represents a family‐level feature of Serpulidae. The new species also exhibits frequent asexual reproduction by budding and anterior regeneration, extending known life‐history diversity in the group. Together, these findings enhance our understanding of serpulid systematics, life history, and mitochondrial evolution, underscoring the value of integrative approaches in uncovering annelid biodiversity from underexplored regions.

## Graphical abstract

We describe *Salmacina stellaebayensis* n. sp. from Mauritania's Baie de l'Étoile and provide the first complete mitochondrial genome for the genus *Salmacina*. The species forms distinctive macroid colonies in *Cymodocea nodosa* seagrass meadows, offering new insights into serpulid life history and habitat specialization. Phylogenetic and mitogenomic analyses clarify relationships within the family and highlight the remarkable variability of mitochondrial gene order across Serpulidae.

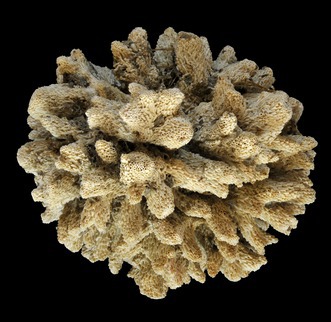

## 1 Introduction

Patterns of marine biodiversity are strongly influenced by uneven sampling intensity, with mid‐latitude regions receiving most attention while vast stretches of the African Atlantic coast remain poorly explored (Thyrring et al. 2024). The polychaete fauna of the nearby archipelagos of Madeira and the Canary Islands was studied in detail in the seminal works of Paul Langerhans in the late 19th century (Hausen 1987; Langerhans 1879, 1880, 1881), providing the most geographically relevant baseline for comparison with the Mauritanian fauna. In contrast, the Mauritanian coastline has seen little systematic investigation, and its polychaete diversity remains poorly documented (Gillet 1990; Gillet and Mouloud 2020; Knorrn et al. 2025). During recent sampling along this coast, a serpulid worm forming striking star‐shaped pseudo‐colonies was collected from Baie de l'Étoile (“Bay of the Star”). While the name of the locality may be a coincidence, the appearance of the colonies resonates with the bay's evocative name.

The specimens could be assigned to the *Salmacina*–*Filograna* complex, a group long recognized as taxonomically challenging (Ten Hove and Kupriyanova 2009). Distinctions between *Salmacina* Claparède, 1870 and its close relative *Filograna* Berkeley, 1835 are traditionally based on the presence of opercula, absent in *Salmacina* (e.g., Fauvel 1927). However, subsequent studies reported operculate and non‐operculate forms co‐occurring in single populations, raising doubts about the diagnostic value of this trait (Kupriyanova and Jirkov 1997; Lehrke et al. 2007). Owing to these difficulties and the limited availability of molecular data, the taxonomy of the complex has remained poorly resolved.

Molecular identification of serpulids is further complicated by technical difficulties: amplification of the COI barcoding marker fails with standard primers, resulting in major gaps in reference databases (Capa et al. 2021; Kupriyanova et al. 2008). At the same time, Serpulidae are remarkable for their extensive plasticity in mitochondrial gene order (Struck et al. 2023; Sun et al. 2021), making them a useful model for studying structural evolution of animal mitogenomes. Yet despite this potential, complete mitochondrial genomes remain available for only a handful of serpulid genera, and none have so far been reported for *Salmacina*.

In this study we describe *Salmacina stellaebayensis* n. sp. from Baie de l'Étoile, Mauritania, using an integrative approach that combines detailed morphological observations with next‐generation sequencing. We provide the first complete mitochondrial genome for the genus *Salmacina*, analyze its phylogenetic placement within Serpulidae, and assess mitogenome evolution across all currently available serpulid mitogenomes. Together, these results (i) clarify the taxonomic placement of the Mauritanian specimen, (ii) provide novel genomic resources for serpulid systematics, and (iii) contribute to a better understanding of the evolution of mitochondrial gene order within this morphologically and genomically diverse annelid family, and (iv) offer new insights into the distinct life history and macroid formation of the species in *Cymodocea nodosa* Ascherson, 1870 meadows.

## 2 Methods

### 2.1 Specimen Collection and Preservation

*Salmacina stellaebayensis* n. sp. pseudo‐colonies were hand collected during three different field trips between 2022 and 2024 in 2–3 m depth at Baie de l'Etoile in northern Mauritania (21.024875, −17.00718). The sampling was conducted during low tide along seagrass *Cymodocea nodosa* Ascherson, 1870 beds located in the hydrodynamic shadow of a small rocky island within the Baie de l'Etoile. Larger tube aggregations were preserved in formalin and subsequently transferred to 70% ethanol, while smaller fragments were directly transferred to 96% ethanol for molecular studies.

### 2.2 Morphology

Specimens were gently extracted from their tubes by carefully breaking the tube walls open with fine forceps. For scanning electron microscopy (SEM), well‐preserved animals were selected, dehydrated through an ascending ethanol series, and subjected to hexamethyldisilazane (HMDS) drying: 15 min in 80% ethanol, 15 min in 90% ethanol, 15 min in 95% ethanol, twice 15 min in 100% ethanol, 20 min in 1:2 [HMDS:EtOH 100%], 20 min in 1:1 [HMDS:EtOH 100%], 20 min in 2:1 [HMDS:EtOH 100%], and 20 min in 100% HMDS, with a final change to HMDS before air drying overnight under a fume hood. Dried specimens were mounted on aluminum stubs, sputter‐coated with gold–palladium (Au‐Pd) alloy to enhance conductivity, and examined with a Hitachi TM400 plus Tabletop SEM.

### 2.3 Genome Assembly and Annotation

For molecular analyses, *Salmacina stellaebayensis* n. sp. samples preserved in 96% and stored at −24°C were used, with whole animals processed due to their small size (1–2 mm in total length). The DNA extraction followed the DNeasy Blood and Tissue Kit protocol (Qiagen Inc.), and DNA quantity was determined using the Qubit Fluorometer (Invitrogen, Quant‐iT dsDNA HS Assay) (yield: 5.59 ng/μL). The DNA extraction was shipped to Novogene Europe for wholegenome library preparation and Illumina sequencing. Libraries with a targeted insert size of 300 bp were prepared and sequenced as 150 bp paired‐end reads on a NovaSeq X Plus platform, generating a total of 24,709,305 raw read pairs. All raw genome skimming data are available in the SRA Archive under the BioProject accession number PRJNA1348991. Reads were subsampled by 50%, yielding 12,353,764 pairs for assembly and further analysis. The complete circular mitogenome of *S. stellaebayensis* n. sp. was assembled from raw reads using MitoZ v3.6 (Meng et al. 2019) without a seed sequence and annotated with MITOS2 v2.2.9 (genetic code 5, RefSeq63 Metazoa) (Donath et al. 2019). Annotation results from both pipelines were manually curated and validated in Geneious Prime v2025.2.1 (Biomatters). Missing or ambiguous protein‐coding genes (PCGs) and rRNAs were manually annotated via cross‐species sequence comparison. Within MitoZ, quality trimming and filtering of raw reads were performed with fastp (Chen 2023). The filtered reads were further assembled with SPAdes v3.15.0 (Prjibelski et al. 2020) using the standard parameters. The resulting scaffolds were used as a BLAST database to recover phylogenetically informative nuclear markers, including the complete ribosomal cluster comprising 18S rRNA, ITS1, 5.8S rRNA, ITS2, and 28S rRNA (GenBank: PX614073). The mitogenome map was drawn using Organellar Genome DRAW (ORGDRAW) (Lohse et al. 2013). AT and GC skews were calculated using the formulas of Perna and Kocher (1995): AT skew = (A−T)/(A+T), GC skew = (G−C)/(G+C). In this context, negative skew values indicate an enrichment of thymine or cytosine on the coding strand, whereas positive values reflect a higher proportion of adenine or guanine.

### 2.4 Phylogenetic Analyses

All available 18S rRNA sequences of *Salmacina* and *Filograna* species were retrieved from GenBank, together with *Protis hydrothermica* (used as outgroup following Kupriyanova et al. 2023), and complemented by the sequence generated in this study (Table S1). Sequences were aligned in Geneious Prime v2025.2.1 using MAFFT v7.490 (Katoh et al. 2002; Katoh and Standley 2013) with default parameters. Maximum likelihood (ML) phylogenetic reconstruction was performed in IQ‐TREE v3.0.1 (Wong et al. 2025), using the substitution model selected by ModelFinder (Kalyaanamoorthy et al. 2017). The K2P + G4 model was identified as the best fit according to the Bayesian Information Criterion (BIC). Branch support was estimated from 1000 ultrafast bootstrap replicates (Hoang et al. 2017).

All available serpulid mitogenomes and the whole mitogenome of *Chone infundibuliformis* Krøyer, 1856 (Sabellidae) as the outgroup were retrieved from GenBank (Table S1). If an annotation was lacking on GenBank, mitogenomes were annotated as described for *S. stellaebayensis* above. Sequences of 12 mitochondrial PCGs and two rRNAs were aligned using MAFFT v. 7.490 in Geneious Prime v2025.2.2 with default parameters. All 14 alignments were concatenated with FASconCAT‐G (Kück and Longo 2014). The best fitting model for each partition was chosen using ModelFinder (Table S2), and a maximum likelihood tree was constructed in IQ‐Tree v. 3.0.1. Branch support was obtained from 1000 ultrafast bootstrap replicates.

## 3 Results

### 3.1 Species Description

Class Polychaeta Grube, 1850.

Subclass Sedentaria Lamarck, 1818.

Order Sabellida Levinsen, 1825.

Family Serpulidae Rafinesque, 1815.

Subfamily Filograninae Rioja, 1923.

Genus *Salmacina* Claparède, 1870.

*Salmacina stellaebayensis* n. sp.

LSID: urn:lsid:zoobank.org:act:0FE30E51‐1E9D‐49FC‐9D73‐E1C6DF682610.

**Material examined:**

**Holotype**.

- SMF 33427 (on SEM stub), *Salmacina stellaebayensis* n. sp., collected from Baie de l'Étoile, Nouadhibou, Mauritania (Station BdE‐63; 21°1.4925′ N, 17°0.4308′ W), 17 February 2023; leg. Alex H. Knorrn.

**Paratypes**.

- SMF 33426, 33425, 33424, 33423, 33422 (on SEM stubs), same data as holotype.
- SMF 33345 and SMF 33392 (pseudo‐colonies)
  – SMF 33345: Station BdE‐63 (21°1.4925 N, 17°0.4308′ W), 17 February 2023.
  – SMF 33392: Station BdE‐33 (21°1.179450′ N–21°1.331992′ N, 17°0.323399′ W–17°0.387188′ W), 2 August 2022.
- IMROP 14 (pseudo‐colonies), same locality as SMF 33345, deposited at IMROP (Institut Mauritanien de Recherches Océanographiques et des Pêches).
- SMF 33390 (isolated tissue sample), same locality, stored at −20°C for molecular analyses.

**Repositories**.

SMF—Senckenberg Natural History Museum and Research Institute, Frankfurt, Section Marine Invertebrates II.

IMROP—Institut Mauritanien de Recherches Océanographiques et des Pêches, Nouadhibou, Mauritania.

**Diagnosis:** Tubes fragile, forming extensive star‐shaped pseudo‐colonies composed of interwoven, encrusting tubes. Radiolar crown with four pairs of slender radioles lacking opercula; radioles not inflated distally. Collar chaetae of fin‐and‐blade type, coarsely toothed, with large distal teeth on fin decreasing progressively in size proximally and separated from finely denticulated blade by distinct V‐shaped gap. Thoracic uncini rasp‐shaped, with 6–7 transverse rows of teeth and 2–5 teeth per row; abdominal uncini broader, bearing 9–11 rows with 5–8 teeth.

**Description:**

**FIGURE 1:**
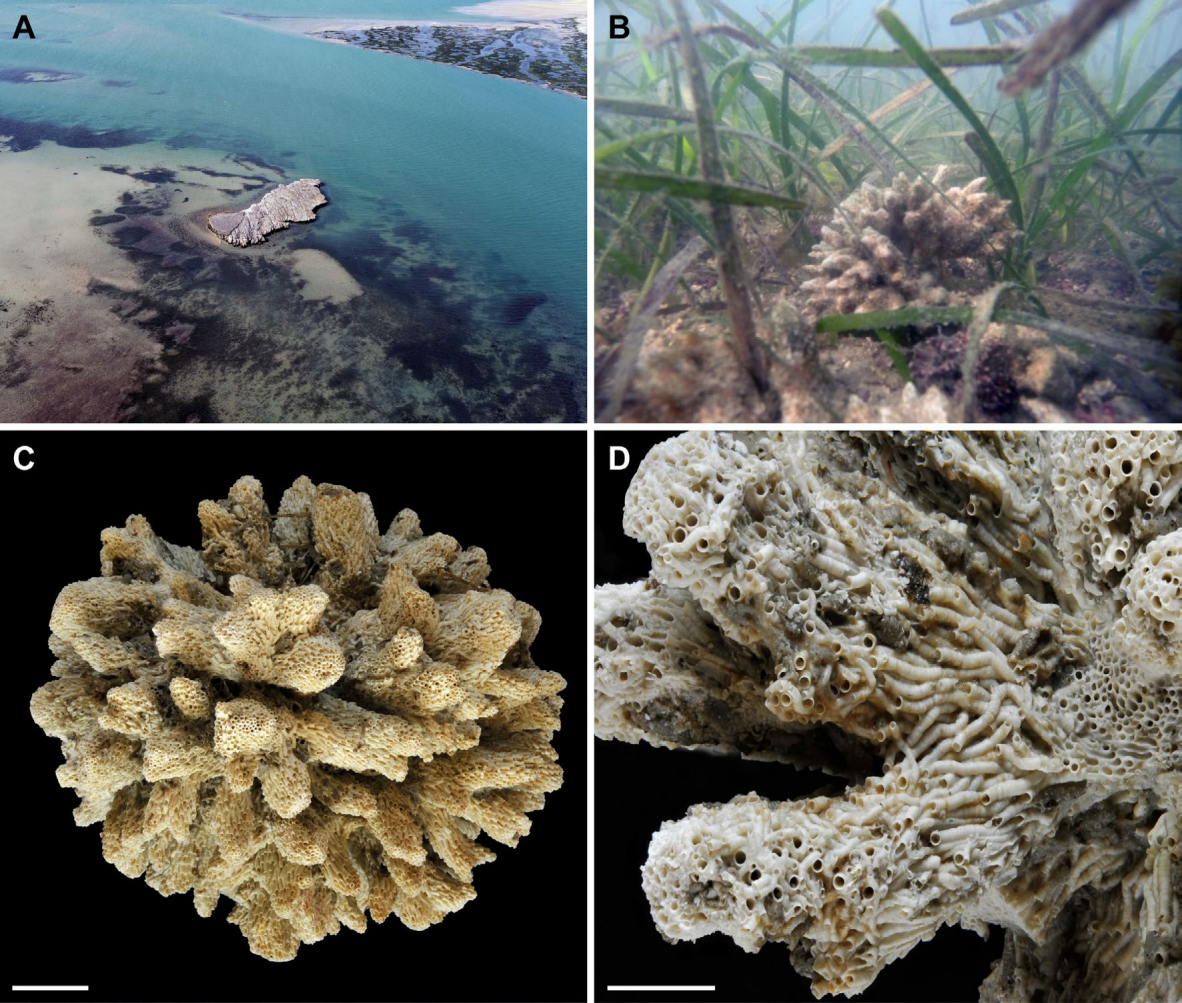
Habitat, pseudo‐colonies, and habitus of *Salmacina stellaebayensis* n. sp. (A) Aerial view of Baie de l'Étoile and the rocky island located in the centre of the embayment, the eponymous type locality and sampling site of the new species, showing extensive seagrass and maerl beds. (B) Underwater photograph of a *Salmacina* pseudo‐colony growing within a *Cymodocea nodosa* bed. (C) Overview of a star‐shaped pseudo‐colony. (D) Detail of interwoven tubes within the aggregation. Scale bars: C = 1 cm; D = 0.5 cm.

**Tube:** Fragile, forming large star‐shaped pseudo‐colonies up to 17 cm in diameter, composed of numerous interwoven tubes encrusting one another (Figure 1B,C). Individual tubes white to pale brown, smooth, with faint transverse ridges. Tube mouth circular, occasionally half‐circular where tubes are densely clustered (Figure 1D). Tubes straight, without evidence of spiral coiling.

**Body size:** *Holotype—*Total body length, including the radiolar crown, 1.8 mm. Total length ranges from 1 to 2.3 mm (*n* = 13; mean = 1.5 ± 0.5). Extended body length in some individuals may reflect artifacts from extraction.

**FIGURE 2:**
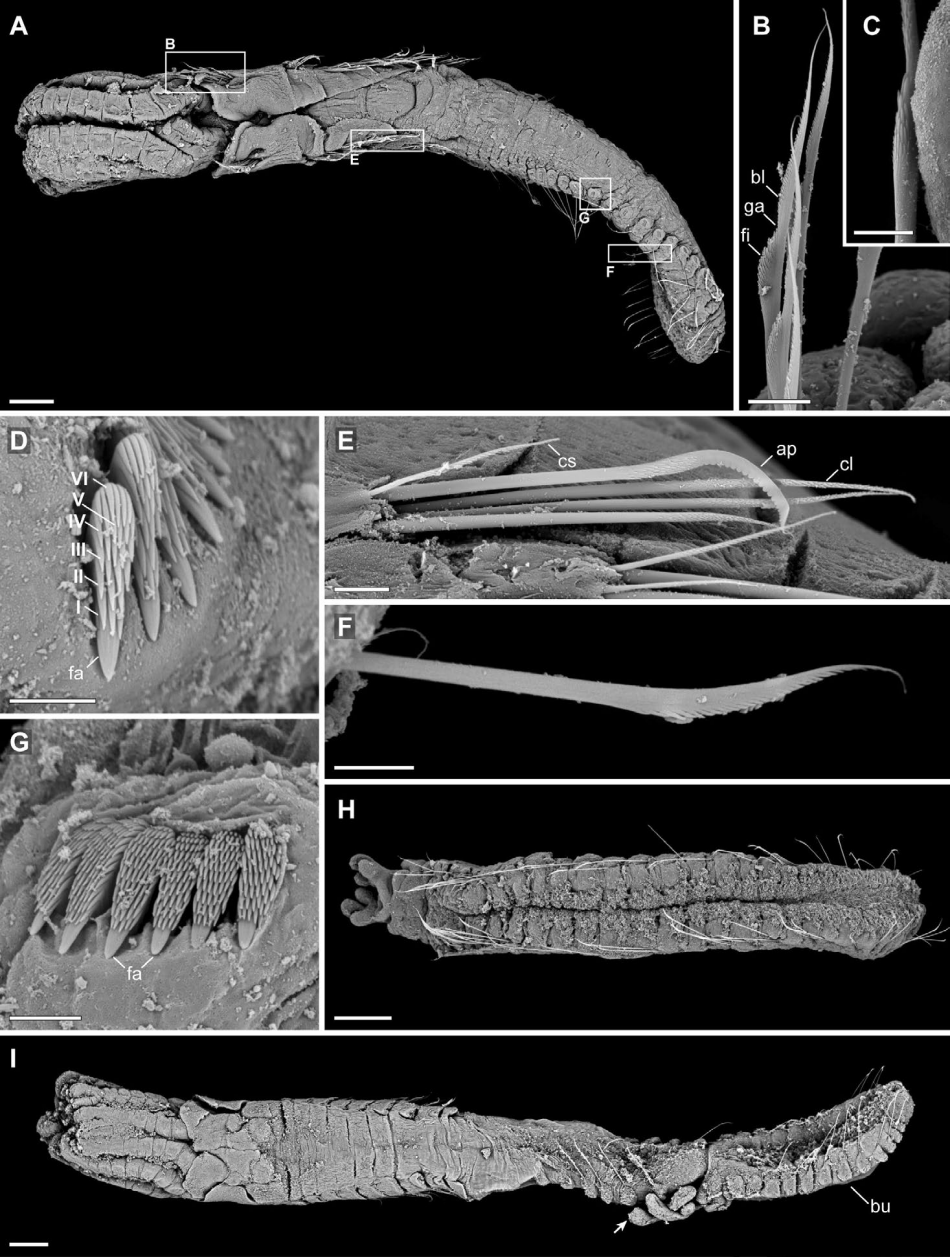
Scanning electron micrographs (SEM) of *Salmacina stellaebayensis* n. sp. (A) Whole specimen, dorsal view (habitus), holotype SMF 33427. (B) Fin‐and‐blade type collar chaetae from the first chaetiger, SMF 33423. (C) Detail of denticles in the fin of the special collar chaetae, SMF 33426. (D) Rasp‐shaped thoracic uncini from the fourth unciniger; Roman numerals indicate the number of horizontal tooth rows above the fang, SMF 33423. (E) Anterior rasp‐shaped abdominal chaetae from the third unciniger, SMF 33425. (F) Limbate chaetae from the 18th abdominal unciniger, SMF 33424. (G) Abdominal uncini from the 13th unciniger, SMF 33423. (H) Ventral view of a specimen showing only abdominal segments and regeneration of the radiolar crown, SMF 33424. (I) Ventrolateral view of a specimen undergoing asexual reproduction; arrow indicates the rudiment of the radiolar crown, SMF 33422. a*p*, *Apomatus* chaetae; *bl*, blade; *bu*, bud; *cl*, long capillary chaeta; *cs*, short capillary chaeta; *fa*, fang; *fi*, fin; *ga*, gap. Scale bars: A, H, I = 100 μm; B–G = 10 μm.

**Radiolar crown:** Lacking opercula. Radiolar crown with four pairs of radioles arranged in a single circle; radioles slender, of uniform diameter along entire length, with tips not inflated or enlarged in preserved material (Figure 2A). Interradiolar membrane absent.

**Collar:** Trilobed, comprising two well‐developed latero‐dorsal lobes (Figure 2A) and a single medio‐ventral lobe forming a lip‐like projection. Collar length one‐third to one‐half that of the radioles. Chaetae of the collar include both fin‐and‐blade type (Figure 2B) and smaller limbate forms. Blade with minute denticles, separated from a similarly denticulated fin by a narrow, V‐shaped gap devoid of denticles (Figure 2B,C). Denticles on fin increasing in size distally (Figure 2C).

**Thorax:** *Holotype—*Consisting of eight chaetigers; collar bearing collar chaetae followed by seven uncinigers. Number of thoracic chaetigers varies from 7 to 10 (*n* = 19, mean = 8.05 ± 0.62). Well‐developed thoracic membranes extending to the last thoracic unciniger not forming an apron ventrally. Dorsal chaetae capillary, of two distinct sizes (long and short). *Apomatus*‐chaetae appearing from second unciniger onwards (Figure 2E). Both capillary and *Apomatus*‐chaetae with minute denticles; latter with blunt teeth along blade. Uncini present from chaetiger 2 onwards, rasp‐shaped, with 6–7 rows of teeth above a large pointed anterior fang; each horizontal row bearing 2–4 proximal teeth increasing to 4–5 distally (Figure 2D). Achaetous zone between thorax and abdomen absent.

**Abdomen:** *Holotype—*comprising 27 segments. Abdominal segment counts range from 15 to 27 (*n* = 12, mean = 19.83 ± 4.04). Each segment with a flat geniculate chaeta having a slender tip and narrow distal zone bearing small teeth (Figure 2F). Abdominal uncini rasp‐shaped, broader than thoracic ones, with a stout, pointed fang and 9–11 rows of curved teeth above the fang (Figure 2G). Proximal to the fang, 5–6 curved and pointed teeth, increasing centrally to a maximum of eight. Dental formula P:5:6:6:7:7:8:8:7:6:5. Pygidium bilobed.

**Asexual reproduction:** Observed in paratypes only. Budding documented in at least three specimens (Figure 2I). Developing individuals display an incipient radiolar crown prior to thoracic development. Buds consisting only of abdominal segments already bear well‐developed radiolar crowns (Figure 2I). One specimen shows regeneration of a radiolar crown while retaining only collar and abdominal segments (Figure 2H).

**Etymology:** The species epithet *stellaebayensis* is a compound derived from the Latin *stella* (star) and *bayensis* (from the bay), referring to Baie de l'Étoile (“Bay of the Star”) in Mauritania, the type locality of the species. The name also alludes to the star‐shaped pseudocolonies formed by this species.

**Ecology & Habitat:** The species occurs in shallow coastal waters (0.5–2 m depth), forming striking star‐shaped pseudo‐colonies, or so‐called macroids, as tubes grow around the rhizomes of the seagrass *Cymodocea nodosa* Ascherson, 1870. Many macroids retain an open central cavity where the rhizome was originally located. When the rhizomes detach, the colonies become free and settle on the seafloor, where they continue to grow as encrusting, star‐shaped aggregations. The colonies are occasionally consumed by hermit crabs of the species *Pseudopagurus granulimanus* (Miers, 1881). The sediment underlying the *Cymodocea* meadow consists of coarse shelly gravel and maerl.

**Remarks:** Among described congeners, *Salmacina setosa* Langerhans, 1884 is geographically the closest to *S. stellaebayensis* n. sp. and therefore the most relevant species for comparison. Both occur along the eastern Atlantic, with the type locality of *S. setosa* (Madeira) lying only a few hundred kilometers north of Mauritania. However, published records attributed to *S. setosa* differ greatly in geography, depth, and morphology, casting doubt on its taxonomic coherence (Table S3).

The original description of *S. setosa* by Langerhans, 1884 was brief, with only 8 sentences, and illustrated only schematically (Figure S1A), and no further descriptions from Madeira exist. Subsequent accounts by Southward (1963) and Nogueira and ten Hove (2000) were based on material from bathyal depths, apparently following Langerhans' vague reference to “greater depths.” Yet in an earlier work, Langerhans (1879) defined this as 54–180 m, considerably shallower than those later reports. In contrast, *S. stellaebayensis* was collected from shallow water (0.5–2 m).

Morphological discrepancies among the available descriptions of *S. setosa* are equally striking. Langerhans (1884) and Southward (1963) both described six thoracic chaetigers, whereas the Mauritanian specimens show seven to ten. Abdominal segment counts also vary widely among published records (Table S3). Although the number of thoracic chaetigers is typically stable in Serpulidae (usually seven), it can vary from six to twelve within *Filograna*/*Salmacina* due to asexual reproduction (Kupriyanova et al. 2006) and is therefore of limited diagnostic value.

Chaetal features offer more decisive distinctions. *S. stellaebayensis* has collar chaetae of fin‐and‐blade type with coarse dentition; large distal teeth becoming smaller proximally and separated by a distinct V‐shaped interdentate gap, whereas *S. setosa* has been described as having either seven large teeth (Nogueira and ten Hove 2000) or finely serrated edges (Southward 1963). The number of teeth in thoracic and abdominal uncini, considered diagnostic in Serpulidae (Capa et al. 2021), also differs between *S. stellaebayensis* and all specimens referred to *S. setosa* . Moreover, *S. stellaebayensis* forms distinctive star‐shaped pseudo‐colonies, a feature not reported for any other *Salmacina* species.

Langerhans (1884) himself suggested that *S. setosa* might represent a juvenile form of another *Salmacina* species, noting a chaetal fascicle between thorax and abdomen later interpreted as larval chaetae (Southward 1963), a structure absent in the Mauritanian material. The specimens attributed to Langerhans' *S. setosa* , housed in the Natural History Museum Vienna (NHMV 2526), consist of two degraded slides that were never formally designated as types (pers. comm. Chloé Stévenne, NHMV). Although some structures correspond to Langerhans' drawings, the material is too deteriorated for meaningful comparison (Figure S1).

Taken together, the morphological and ecological differences clearly distinguish *S. stellaebayensis* from the geographically proximate *S. setosa* and other congeners (see Table S3). At the same time, inconsistencies among records attributed to *S. setosa* suggest that this name may encompass multiple species and warrant comprehensive re‐examination of topotypical material from Madeira.

### 3.2 Mitochondrial Genome and Supporting Nuclear Data

**FIGURE 3:**
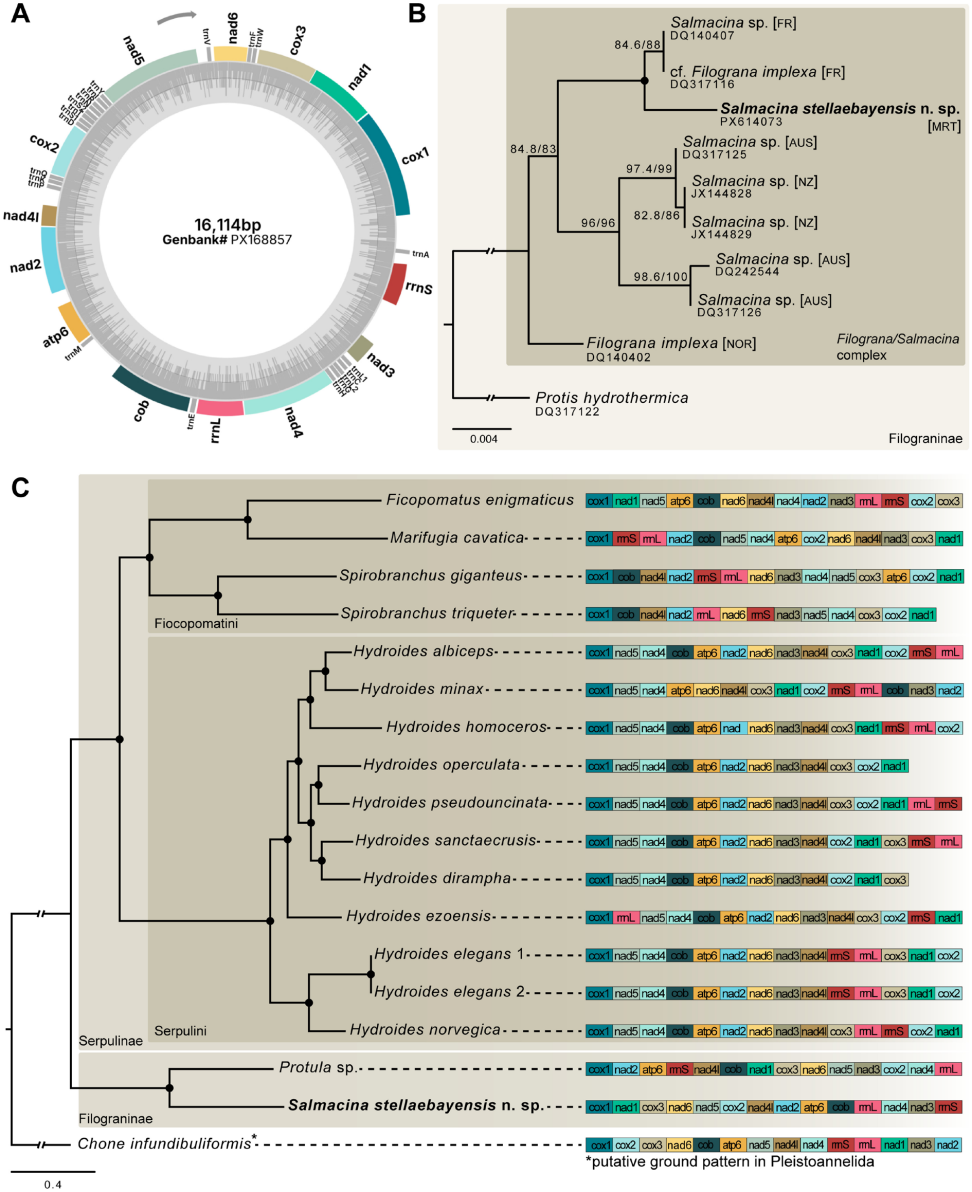
Mitochondrial genome organization and phylogenetic placement of *Salmacina stellaebayensis* n. sp. (A) Circular map of the mitochondrial genome of *S. stellaebayensis*, showing genome size (bp) and GenBank accession number in the center. (B) Maximum‐likelihood phylogenetic tree based on 18S rRNA sequences of *Salmacina* and *Filograna* species. *Protis hydrothermica* was used as an outgroup. Branch support values are shown where below 99. (C) Maximum‐likelihood phylogenetic tree based on concatenated mitochondrial protein‐coding genes (12 PCGs) and two rRNAs from complete mitogenomes retrieved from GenBank. *S. stellaebayensis* n. sp. is highlighted in bold. Branch support values are shown only if below 99.5. The gene order of PCGs and rRNAs is indicated on the right; the putative ground pattern for Pleistoannelida is represented by the outgroup *Chone infundibuliformis* , with atp8 omitted for clarity.

The mitochondrial genome of *Salmacina stellaebayensis* n. sp. is 16,144 bp in length and has been deposited in GenBank under accession number PX168857 (Figure 3A). Annotation identified 12 protein‐coding genes, two rRNAs, and 22 tRNAs; however, atp8 was not detected by either automated annotation or manual cross‐species validation. The gene order is shown in Figure 3A,C, with all protein‐coding genes transcribed from the same strand.

The overall nucleotide composition is 23.1% A, 12.8% C, 25.7% G, and 38.4% T, resulting in an AT‐rich genome (61.5%) and a GC content of 38.5%. The genome exhibits a negative AT skew (−0.24) and a positive GC skew (0.33).

For comparative analyses, 13 additional serpulid mitochondrial genomes were retrieved and re‐annotated from GenBank (Table S1). As in *S. stellaebayensis*, atp8 could not be identified in any of the analyzed genomes and was therefore excluded from subsequent phylogenetic analyses. In *Spirobranchus triqueter* (Linnaeus, 1758) and *Hydroides dirampha* Mörch, 1863, atp6 was also missing, and annotations of *H. dirampha* and *H. pseudouncinata* Zibrowius, 1968 lacked both the small (rrnS) and large (rrnL) rRNA genes.

In addition to the mitochondrial genome, the complete ribosomal cluster comprising 18S rRNA, ITS1, 5.8S rRNA, ITS2, and 28S rRNA was recovered for *Salmacina stellaebayensis* n. sp., and the corresponding sequences have been deposited in GenBank (Table S1). To assess the phylogenetic position of the new species within *Salmacina*/*Filograna*, all available 18S rRNA sequences of *Salmacina* and *Filograna* species, as well as *Protis hydrothermica* (used as outgroup following Kupriyanova et al. 2023), were analyzed. The resulting maximum‐likelihood tree (Figure 3B) recovered *Filograna implexa* (DQ140402) from Norway in a clade with *Salmacina* spp. and another sequence identified as *Filograna implexa* (DQ317116), which is identical to a *Salmacina* species from France. Independent information on the original specimen indicates that this sequence was misidentified at the time of submission (Kupriyanova, *pers. comm.). Together*, these Atlantic sequences, including *S. stellaebayensis* n. sp., form a well‐supported clade that is sister to a second clade composed of *Salmacina* species from Australia and New Zealand.

### 3.3 Phylogeny and Mitogenome Evolution

Maximum‐likelihood (ML) analysis based on concatenated alignments of 12 mitochondrial protein‐coding genes and two rRNAs recovered Serpulidae as monophyletic and revealed two well‐supported subclades (Figure 3C). The first included *Protula* sp. and *Salmacina stellaebayensis* n. sp. as sister taxa, while the second comprised two major lineages: one consisting exclusively of *Hydroides* Gunnerus, 1768 species, recovered as monophyletic, and the other including *Spirobranchus* species together with *Marifugia cavatica* Absolon & Hrabe, 1930 and *Ficopomatus enigmaticus* (Fauvel, 1923). These results are consistent with and support the monophyly of both Filograninae Rioja, 1923 and Serpulinae Rafinesque, 1815, and also support the monophyly of Ficopomatini Pillai, 1960 within Serpulinae.

Within *Hydroides*, two well‐supported subclades were recovered: one grouping *H. norvegica* Gunnerus, 1768 with *H. elegans* (Haswell, 1883), and the other comprising all remaining *Hydroides* species as sister to *H. ezoensis* Okuda, 1984. Both higher‐ and species‐level relationships were strongly supported by bootstrap and SH‐aLRT values, with minimum bootstrap and SH‐aLRT support values of 99.6 and 98, respectively.

Mitochondrial gene order varied markedly among the 17 serpulid genomes analyzed, particularly in the positions of ribosomal RNA genes, which differed both between subfamilies and among species within the same genus (Figure 3C). With the exception of *Hydroides*, each serpulid species exhibited a unique gene arrangement. Within *Hydroides*, the arrangement of five consecutive protein‐coding genes (cox1–nad5–nad4–cob–atp6) was conserved, although *H. ezoensis* and *H. minax* (Grube, 1878) showed deviations from this pattern. No gene block was conserved across all examined serpulid mitochondrial genomes (Figure 3C).

## 4 Discussion

### 4.1 Life History

The branching colonies of *Salmacina* sp. from Mauritania appear to result from a combination of asexual budding and gregarious larval settlement, as previously reported for *Salmacina* and other serpulids (Ten Hove 1979; Nishi 1993; Nishi and Nishihira 1994; Kupriyanova et al. 2001). Sexual reproduction in polychaetes encompasses a wide diversity of life‐history strategies. In contrast, asexual reproduction is comparatively less common across the group but occurs relatively frequently in serpulids (Kupriyanova et al. 2001; Nishi and Nishihira 1994). Unlike free‐living or mobile sedentary polychaetes, serpulids lack reproductive traits such as swarming, epitoky, or direct copulation. Their obligately sessile lifestyle likely constrains reproductive biology, favoring alternative strategies such as clonal propagation by budding and intratubal brooding (Kupriyanova et al. 2001, 2019).

Most individuals examined originated from the peripheral portions of the pseudo‐colonies, which typically harbor a higher proportion of asexually reproducing worms compared with the basal and central regions (Nishi and Nishihira 1994). Asexual budding was frequently observed and follows the developmental sequence described by Nishi (1993). The earliest visible sign of budding is the appearance of branchial rudiments near the last abdominal unciniger, which gradually develop into a nearly complete radiolar crown before thoracic membranes and chaetigers appear. In the *Salmacina stellaebayensis* n. sp., buds often possessed well‐developed radiolar crown rudiments but lacked thoracic segments, confirming this pattern.

Intratubal egg brooding has been reported in *Salmacina* by Nishi (1993) and Pernet (2001), but no eggs were observed in the present material. This absence likely reflects the timing of collection rather than the absence of brooding altogether. Pernet (2001), for instance, observed no sexual reproduction over 6 months, whereas adults consistently produced one to four buds per month, indicating that asexual reproduction predominates but does not exclude occasional sexual reproduction.

In addition to budding, anterior regeneration was also recorded, with worms regenerating lost thoracic segments and radiolar crowns. Although regeneration has not been documented previously in *Salmacina*, it is well known in other serpulids, such as *Turbocavus secretus* (Prentiss et al. 2014), and is considered a common feature within Serpulidae (Bely 2006).

Of particular note is the primary attachment of *Salmacina* pseudo‐colonies to the rhizome network of the seagrass *Cymodocea nodosa*. As a result, many well‐preserved and later exposed macroids exhibit a central hole marking the original site of rhizome growth. Subsequent rearrangements, driven by tidal currents, wave action, and the activity of large decapods such as *Pseudopagurus granulimanus* or fish, appear to promote the separation and further growth of individual macroid fragments. This process likely explains both the characteristic star‐shaped morphology of the macroids and the considerable variation in colony form, as no two *Salmacina* macroids are alike. Reports of *Salmacina* species growing on seagrass rhizoids are rare, with *S. incrustans* Claparède, 1870 being one of the few documented examples, living on *Posidonia oceanica* (Linnaeus) Delile, 1813 rhizomes (Piazzi et al. 2016). Other instances of fixed and gregarious settlements within the *Salmacina*/*Filograna* complex have been reported from underwater freshwater springs in the northern Adriatic Sea (Cocito et al. 2004) and from the Gulf of Oman (Samimi‐Namin et al. 2022).

### 4.2 Mitogenome Composition and Annotation

In the circularized mitogenome of *Salmacina stellaebayensis* n. sp., all tRNA and rRNA genes could be annotated, whereas one protein‐coding gene (atp8) was not detected. The absence of atp8 has been reported previously in annelids, first in *Spirobranchus giganteus* (Pallas, 1766) (Seixas et al. 2017), and also in several other metazoan phyla including Acoelomorpha (Mwinyi et al. 2010), Chaetognatha (Shen et al. 2016), Mollusca (Stöger and Schrödl 2013), Platyhelminthes (Aguado, Grande, et al. 2016), and Porifera (Lavrov et al. 2016). Nevertheless, atp8 is part of the ancestral mitochondrial gene set of Annelida (Weigert et al. 2016), and its apparent absence in many lineages likely reflects annotation difficulties rather than genuine gene loss.

Annotation of atp8 is challenging because of its short length (typically around 160 bp) and high sequence divergence, which complicate homology assessment. Automated pipelines often fail to detect this gene (Bernt, Braband, et al. 2013; Feng and Sun 2025), and even manual searches by cross‐species comparison were unsuccessful in the present dataset. However, Sun et al. (2021) demonstrated that putative atp8 homologs can be recovered in *Hydroides* by predicting all open reading frames (ORFs), translating them, and screening amino acid sequences against a profile hidden Markov model (HMM) constructed from ATP8 alignments. The apparent absence of atp8 in the present mitogenome is therefore interpreted as an annotation limitation rather than evidence for gene loss, and does not affect downstream analyses.

The complete mitogenome of *S. stellaebayensis* is slightly larger than the median size of annelid mitogenomes (15,356 bp) reported by Struck et al. (2023). Large mitogenome sizes are not unusual within Serpulidae; *Hydroides* species reach up to 25,087 bp (Sun et al. 2021), and *Spirobranchus giganteus* possesses a mitogenome of 22,058 bp (Seixas et al. 2017).

### 4.3 Molecular Phylogenetics

Previous phylogenetic studies of Serpulidae have primarily relied on alignments of the nuclear 18S and 28S rRNA genes, complemented by parsimony analyses of morphological characters (Kupriyanova et al. 2006; Lehrke et al. 2007). More recently, Kupriyanova et al. (2023) expanded these datasets by including additional markers such as H3 and COB, adopting an integrative approach to resolve serpulid relationships. Their results demonstrated that morphological features traditionally used in serpulid taxonomy, particularly the operculum, are poor predictors of evolutionary relationships.

The 18S rRNA phylogeny (Figure 3B) recovered *Filograna implexa* as sister to a clade comprising *Salmacina stellaebayensis* and other *Salmacina* species, supporting a close relationship between the two genera but not conclusively resolving their boundaries. This result aligns with previous molecular studies that have consistently recovered *Filograna* and *Salmacina* as closely related lineages (Kupriyanova et al. 2006, 2023; Lehrke et al. 2007). However, the available sequence data remain limited, and the lack of verified operculate *Filograna* specimens precludes firm conclusions regarding the monophyly or distinctiveness of either genus. Additional genomic data from morphologically confirmed *Filograna* and *Salmacina* specimens, ideally including topotypical material, will be essential to clarify the evolutionary relationships within this taxonomically complex group.

The mitochondrial phylogenetic analysis presented here is consistent with the most recent multi‐marker study of serpulids by Kupriyanova et al. (2023), although a phylogeny based solely on mitochondrial markers had not previously been inferred. The ML tree recovered the two primary subfamilies traditionally recognized within Serpulidae, Serpulinae and Filograninae, as well as the tribes Ficopomatini and Serpulini within Serpulinae. While some minor differences in topology are present, these likely result from differences in dataset composition; this study includes 17 complete mitogenomes, whereas Kupriyanova et al. (2023) analyzed mixed markers from 93 specimens. The topology recovered here is also broadly congruent with the recent mitogenomic analysis of *Hydroides* by Sun et al. (2021).

### 4.4 Mitochondrial Gene Order Evolution

Analyses of mitogenomes from 17 serpulid taxa revealed considerable diversity in gene order within Serpulidae (Figure 3C). Among metazoans, mitochondrial gene order is usually highly conserved (Boore and Brown 1998), although exceptions have been documented in several groups such as mollusks (Bernt, Bleidorn, et al. 2013) and crustaceans (Black and Roehrdanz 1998). Because structural variation in mitochondrial genomes can provide an additional source of phylogenetic information beyond sequence data (Boore et al. 1995), understanding these rearrangements is of particular evolutionary relevance.

In annelids, most studies indicate a generally conserved mitochondrial architecture rather than widespread rearrangements (Struck et al. 2023; Weigert et al. 2016). Struck et al. (2023), analyzing 149 annelid mitochondrial genomes, found that 110 retained the predominant gene order, supporting the idea that mitogenomes within Pleistoannelida (Sedentaria + Errantia) are typically stable (Weigert and Bleidorn 2016). Nevertheless, lineage‐specific rearrangements have been increasingly reported, including in Syllidae (Aguado, Richter, et al. 2016; Schwarze et al. 2025), Polynoidae (Hiley et al. 2024; Zhang et al. 2018), Dorvilleidae (Tempestini et al. 2020; Tilic and Rouse 2024), Siboglinidae (Li et al. 2015), and Capitellidae (Su et al. 2025). Within Sabellida, mitochondrial gene order also shows variability. While *Manayunkia occidentalis* Atkinson, Bartholomew & Rouse, 2020 (Fabriciidae), *Chone infundibuliformis* Krøyer, 1856, and *Pseudopotamilla reniformis* (Bruguière, 1789) (Sabellidae) display the putative annelid ground pattern (Figure 3C), more recent studies indicate rearrangements within this clade as well (Putignano and Tilic 2025).

Within Serpulidae, gene order appears especially dynamic. Sun et al. (2021) showed that *Hydroides* species differ markedly from other annelids and even exhibit intrageneric variability. The results of the present study corroborate these findings, supporting the hypothesis that extensive mitochondrial rearrangements may represent a family‐level characteristic in Serpulidae. The underlying causes of this unusual plasticity remain unclear. Because mitochondrial genes are essential for energy metabolism, they are generally expected to be under strong purifying selection, which should constrain genome rearrangements (Zhang et al. 2018). Yet several studies have reported that species with highly rearranged genomes often show elevated substitution rates across protein‐coding genes compared to those with conserved gene order (Su et al. 2025), suggesting a possible link between structural instability and accelerated sequence evolution.

Struck et al. (2023) proposed that biases in nucleotide composition, particularly GC skew, together with high substitution rates and expanded non‐coding regions may promote gene‐order changes in annelids. Notably, *Salmacina stellaebayensis* n. sp. exhibits a negative AT skew and a positive GC skew, whereas most annelids analyzed by Struck et al. (2023) had negative values for both. The same pattern has been observed in *Hydroides*, *Ficopomatus enigmaticus* , and *Spirobranchus giganteus* (Kobayashi et al. 2023; Seixas et al. 2017; Sun et al. 2021). Whether sequence evolution directly drives structural instability, or both arise from a shared underlying factor such as relaxed selection on mitochondrial efficiency, remains unresolved.

Several biological factors have been proposed to explain mitogenome plasticity, but empirical support is limited. Hypotheses include parasitism or inquiline lifestyle (Huč et al. 2024; Tilic and Rouse 2024), elevated oxidative stress (Dowton and Campbell 2001), and adaptation to extreme environments (Wang et al. 2016; Zhang et al. 2018). However, none of these factors have been conclusively linked to genome rearrangements in annelids. The same conclusion currently applies to Serpulidae: their remarkable diversity in gene order is well established, but its underlying evolutionary and functional causes remain to be elucidated.

## 5 Conclusion

The description of *Salmacina stellaebayensis* n. sp. from Mauritania's Baie de l'Étoile expands current knowledge of serpulid diversity along the understudied West African coast and documents a remarkable case of macroid formation associated with seagrass (*Cymodocea nodosa*) meadows. The newly generated mitochondrial and nuclear data provide valuable genomic resources for Serpulidae and reveal a close relationship between *Salmacina* and *Filograna*, although additional molecular evidence is needed to resolve their generic boundaries. The mitogenomic comparisons confirm extensive plasticity in gene order within the family. Together, these findings demonstrate the value of integrative approaches that combine classical morphology, life‐history observations, and genomic data to illuminate evolutionary diversification in Serpulidae and other polychaetes.

## Author Contributions

**Hannah A. Cremer:** formal analysis (lead), investigation (lead), visualization (lead), writing – original draft (lead). **Elena Kupriyanova:** investigation (equal), validation (lead), writing – review and editing (equal). **Alexander H. Knorrn:** investigation (supporting), writing – review and editing (supporting). **Sidi M. M. Moctar:** investigation (supporting), resources (supporting). **Andre Freiwald:** funding acquisition (equal), investigation (equal), resources (equal), writing – review and editing (equal). **Ekin Tilic:** conceptualization (lead), data curation (lead), formal analysis (equal), funding acquisition (lead), supervision (lead), writing – original draft (equal), writing – review and editing (equal).

## Conflicts of Interest

The authors declare no conflicts of interest.

## Supporting information


**Table S1:** Specimens and GenBank accession numbers for mitochondrial genomes and individual gene sequences used in this study. Newly generated sequences are highlighted in bold.
**Table S2:** Substitution models selected by ModelFinder for each mitochondrial locus included in the concatenated mitogenome phylogeny of *Salmacina stellaebayensis* n. sp. and related Serpulidae, based on Bayesian Information Criterion (BIC).
**Table S3:** Comparative morphology and distribution of accepted *Salmacina* species, modified from Nogueira and ten Hove (2000) and supplemented with additional literature records.
**Figure S1:** Historical evidence of *Salmacina setosa* morphology. (A) Original sketches from Langerhans' (1884) description (Tafel XVI, Fig. 40a–g). (B) Microscopic slides of the presumed holotype specimen, Natural History Museum Vienna (collection number 2526). (**C**) Light micrograph of the holotype slide. (D) Detailed light micrograph of the holotype abdomen. Arrows indicate the position of chaetae: red—fascicle of chaetae; black—abdominal chaetae; white—abdominal uncini.

## Data Availability

All newly generated sequence data have been deposited in GenBank. The complete mitochondrial genome of *Salmacina stellaebayensis* n. sp. is available under accession number PX168857, and the raw genome‐skimming reads are deposited under BioProject PRJNA1348991. The complete ribosomal cluster comprising 18S rRNA, ITS1, 5.8S rRNA, ITS2, and 28S rRNA is available under accession number PX614073. All type and voucher specimens, including the holotype (SMF 33427) and paratypes (SMF 33422–33426, 33345, 33390, 33392), are deposited in the Marinve Invertebrates‐II collection of the Senckenberg Natural History Museum and Research Institute Frankfurt (SMF). Additional paratype material (IMROP 14) is housed at the Institut Mauritanien de Recherches Océanographiques et des Pêches (IMROP), Nouadhibou, Mauritania.
