# Supplementary material for: Macroid Formation in Salmacina stellaebayensis n. sp. From Mauritania's Baie de l'Étoile With New Insights on Mitogenome Evolution in Serpulidae (Annelida)
Source: Ecol Evol. 2026 Jan 29;16(2):e73016. doi: 10.1002/ece3.73016 (PMC12853217; doi:10.1002/ece3.73016)
Supplement: Supplementary file 1 — Table S1: Specimens and GenBank accession numbers for mitochondrial genomes and individual gene sequences used in this study. Newly generated sequences are highlighted in bold. Table S2: Substitution models selected by ModelFinder for each mitochondrial locus included in the concatenated mitogenome phylogeny of Salmacina stellaebayensis n. sp. and related Serpulidae, based on Bayesian Information Criterion (BIC). Table S3: Comparative morphology and distribution of accepted Salmacina species, modified from Nogueira and ten Hove (2000) and supplemented with additional literature records. Figure S1: Historical evidence of Salmacina setosa morphology. (A) Original sketches from Langerhans' (1884) description (Tafel XVI, Fig. 40a–g). (B) Microscopic slides of the presumed holotype specimen, Natural History Museum Vienna (collection number 2526). (C) Light micrograph of the holotype slide. (D) Detailed light micrograph of the holotype abdomen. Arrows indicate the position of chaetae: red—fascicle of chaetae; black—abdominal chaetae; white—abdominal uncini. [file ECE3-16-e73016-s001.docx]

**SUPPLEMENT
Table S1.** Specimens and GenBank accession numbers for mitochondrial genomes and individual gene sequences used in this study. Newly generated sequences are highlighted in bold.

| **Species** | **Voucher** | **18S** | **Mitogenome** | **Source** |
| --- | --- | --- | --- | --- |
| *Salmacina stellaebayensis* n. sp. | SMF 33390 | **PX614073** | **PX168857** | This study |
| *Salmacina* sp. [AUS] | SAM E3499 | DQ317126 | – | Kupriyanova et al., 2006 |
| *Salmacina* sp. [AUS] | SAM E3535 | DQ317125 | – | Kupriyanova et al., 2006 |
| *Salmacina* sp. [NZ] | – | JX144828 | – | Smith et al., 2012 |
| *Salmacina* sp. [NZ] | – | JX144829 | – | Smith et al., 2012 |
| *Salmacina* sp. [FR] | ZMA V.Pol. 5244 | DQ140407 | – | Lehrke et al., 2007 |
| cf. *Filograna implexa* [FR] | SAM E3528 | DQ317116 | – | Kupriyanova et al., 2006 |
| *Filograna implexa* [NOR] | ZMA V.Pol. 5239a | DQ140402 | – | Lehrke et al., 2007 |
| *Spirobranchus triqueter* | – | – | OQ729903 | Struck et al., 2023 |
| *Spirobranchus giganteus* | – | – | KX156257 | Seixas et al., 2017 |
| *Hydroides albiceps* | AM W.48807 | – | MW143038 | Sun et al., 2021 |
| *Hydroides dirampha* | HKBU.201301 | – | MW143045 | Sun et al., 2021 |
| *Hydroides elegans* 1 | HKBU 201302 | – | MW143039 | Sun et al., 2021 |
| *Hydroides elegans* 2 | – | – | OM415982 | GenBank |
| *Hydroides ezoensis* | AM W.48019 | – | MW143040 | Sun et al., 2021 |
| *Hydroides homoceros* | AM W.49303 | – | MW143041 | Sun et al., 2021 |
| *Hydroides minax* | AM W.45420 | – | MW143046 | Sun et al., 2021 |
| *Hydroides norvegica* | – | – | MT919975 | Sun et al., 2021 |
| *Hydroides operculata* | AM W.49302 | – | MW143042 | Sun et al., 2021 |
| *Hydroides pseudouncinata* | AM W.47950 | – | MW143043 | Sun et al., 2021 |
| *Hydroides sanctaecrusis* | AM W.46567 | – | MW143044 | Sun et al., 2021 |
| *Marifugia cavatica* | – | – | OZ197803 | GenBank |
| *Protula* sp. | – | – | OX457035 | GenBank |
| *Ficopomatus enigmaticus* | – | – | LC757642 | Kobayashi et al., 2023 |
| *Chone infundibuliformis* | – | – | OQ729908 | Struck et al., 2023 |
|  |  |  |  |  |

**Table S2.** Substitution models selected by ModelFinder for each mitochondrial locus included in the concatenated mitogenome phylogeny of *Salmacina stellaebayensis* n. sp. and related Serpulidae, based on Bayesian Information Criterion (BIC).

| **Gene / Partition** | **Best-fit Model** | **BIC Score** |
| --- | --- | --- |
| 12S rRNA | TIM3+F+G4 | 21168.268 |
| 16S rRNA | TIM3+F+I+G4 | 24603.262 |
| ATP6 | TIM3+F+I+G4 | 20987.676 |
| COX1 | K3Pu+F+I+G4 | 37637.280 |
| COX2 | HKY+F+G4 | 23942.935 |
| COX3 | TPM3u+F+I+G4 | 21779.603 |
| CYTB | K3Pu+F+I+G4 | 31852.769 |
| NAD1 | TIM+F+I+G4 | 24478.554 |
| NAD2 | GTR+F+I+G4 | 32083.497 |
| NAD3 | TPM3u+F+G4 | 9472.883 |
| NAD4L | TPM3u+F+I+G4 | 8518.431 |
| NAD4 | TVM+F+I+G4 | 38446.888 |
| NAD5 | TVM+F+I+G4 | 47565.231 |
| NAD6 | TIM+F+I+G4 | 15643.000 |

**Table S3.** Comparative morphology and distribution of accepted *Salmacina* species, modified from Nogueira & ten Hove (2000) and supplemented with additional literature records.

| **Nominal taxon** | **Source** | **Localities*** | **Depth (m)** | **Pairs of radioles** | **Radiole tip** | **Pinnules with enlarged cells?** | **Teeth in basal fin of collar chaetae** | **Gap** | **No. of thoracic chaetigers** | **Thoracic uncini** | **No. of abdominal chaetigers** | **Abdominal uncini** | **Pros. eyes** |
| --- | --- | --- | --- | --- | --- | --- | --- | --- | --- | --- | --- | --- | --- |
| *S. amphidentata* Jones, 1962 | Nogueira & ten Hove, 2000 | **Port Royal (Jamaica)**; ?South China; Japan | – | 4 | slightly inflated distally | – | regularly increasing in size distally | + | 8 | 8 rows, 4 teeth (3–5) | 17–33 | – | – |
| *S. australis* Haswell, 1884 | Nogueira & ten Hove, 2000 | **Port Jackson (Australia)**; New Zealand | – | 4 | not inflated | – | small denticles only | –? | – | – | 19 | – | – |
| *S. ceciliae* Nogueira & ten Hove, 2000 | Nogueira & ten Hove, 2000 | **Ilha de Alcatrazes (Brazil)** | – | 4 | inflated subdistally | distally | 8 large teeth and many small denticles | + | – | 8–9 rows, 3–4(6) teeth | 28 | – | – |
| *S. chilensis* Gallardo,1969 | Nogueira & ten Hove, 2000 | **off Punta Patache, N. Chile** | bathyal | 10 | not inflated | – | 4-5 large teeth and many small denticles | + | – | – | 36 | – | – |
| *S. dysteri* (Huxley, 1855) | Nogueira & ten Hove, 2000 | **Tenby (UK)**; “worldwide” (probably complex of species) | – | 4 | inflated distally | ? proximally | many small denticles | + | 8 | 7 rows, 2–3 teeth | 27–50 | – | + |
| *S. huxleyi* (Ehlers, 1887) | Nogueira & ten Hove, 2000 | **Tortugas**, Loggerhead Key (Florida); West Indies | – | 4 | inflated distally | – | regularly increasing in size distally | +? | – | ?9 rows | 20 | – | – |
| *S. incrustans* Claparède, 1870 | Nogueira & ten Hove, 2000 | **Naples (Italy)**; “worldwide,” probably complex of species | – | 4 | inflated distally | proximally | 4–6 large teeth | +? | – | many teeth | 40 | – | + |
| *S. piranga* (Grube, 1872) | Nogueira & ten Hove, 2000 | **Estreite, Desterro (Brazil)** | – | 4 | hardly inflated distally | – | 6 large teeth and many small denticles | + | – | 9 rows, 3–5 teeth | 27 | – | + |
| *S. setosa* Langerhans, 1884 | Langerhans, 1884 (orig. descr.) | **Funchal (Madeira)** | 54–180 | 4 | – | – | ? | yes¹ | 6 | 13 rows** | 3–17 | 9 rows** | ? |
| *S. setosa* Langerhans, 1884 | Nogueira & ten Hove, 2000 | W. off England (Great Britain) | bathyal | 4 | ? inflated distally | – | 7 large teeth? | yes | ? | 6–7 rows, 1–3 teeth² | ? | ? | + |
| ?*S. setosa* Langerhans, 1884 | Southward, 1963 | Continental Slope (N Atlantic) | 1390–1775 | 4 | – | – | finely serrated | no | 6 | 8 rows, 2 teeth** | 10–15 | 12 rows, 5–8 teeth** | ? |
| ***S. stellaebayensis* n. sp.** | This study | **Baie de l’Étoile, Nouadhibou (Mauritania)** | 2–3 | 4 | not inflated | – | coarsely toothed, larger distal teeth becoming finer proximally | yes | 8 | 6–7 rows, 2–5 teeth | 13–24 | 9–11 rows, 5–8 teeth | ? |
| *S. tribranchiata* (Moore, 1923) | Nogueira & ten Hove, 2000 | **Santa Rosa Island (California)**; British Columbia; ?Japan | – | 3 | inflated distally | – | “coarsely toothed” | + | – | 8–9 rows, 3–4 teeth | 26 | – | – |
| *type locality in bold, **inferred from drawings  ^1^Langerhans (1884) does not explicitly mention a gap between the fin and blade in his description. However, his drawings depict a V-shaped notch that could be interpreted as such a gap, especially considering that they were based on light microscopy. ^2^Nogueira & ten Hove (2000) describe an unusual variability in number of rows of teeth in their specimens. | | | | | | | | | | | | | |

**
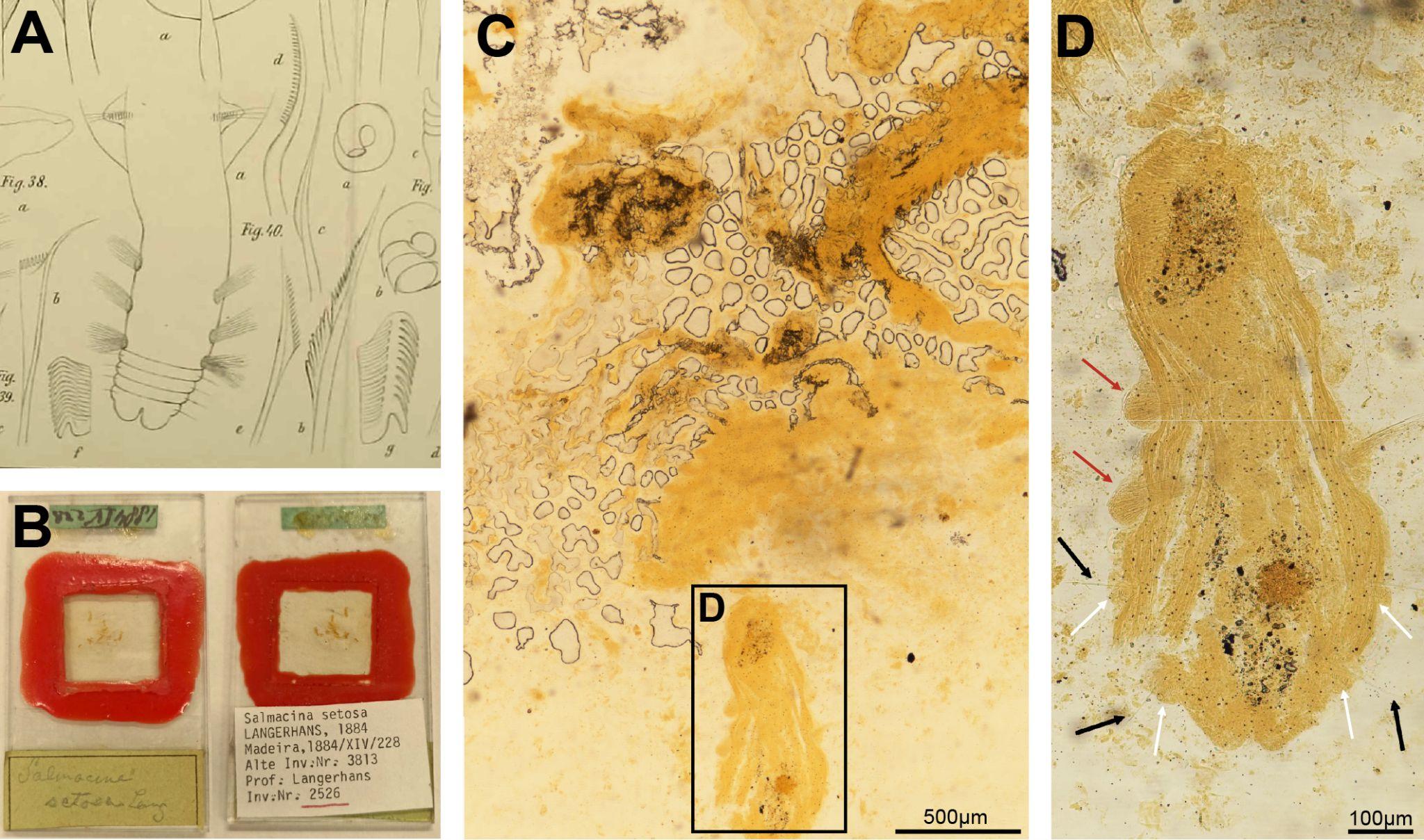


Figure S1.** Historical evidence of *Salmacina setosa* morphology. (**A**) Original sketches from Langerhans’ (1884) description (Tafel XVI, Fig. 40a–g). (**B**) Microscopic slides of the presumed holotype specimen, Natural History Museum Vienna (collection number 2526). (**C**) Light micrograph of the holotype slide. (**D**) Detailed light micrograph of the holotype abdomen. Arrows indicate the position of chaetae: red – fascicle of chaetae; black – abdominal chaetae; white – abdominal uncini.
